# Methylome Profiling of PD-L1-Expressing Glioblastomas Shows Enrichment of Post-Transcriptional and RNA-Associated Gene Regulation

**DOI:** 10.3390/cancers14215375

**Published:** 2022-10-31

**Authors:** Georg Hutarew, Dorothee Hölzl, Tanja Schiefer, Celina K. Langwieder, Beate Alinger-Scharinger, Hans U. Schlicker, Christoph Schwartz, Karl Sotlar, Theo F. J. Kraus

**Affiliations:** 1 Institute of Pathology, University Hospital Salzburg, Paracelsus Medical University, Müllner Hauptstr. 48, A-5020 Salzburg, Austria; 2 Department of Neurosurgery, University Hospital Salzburg, Paracelsus Medical University, Ignaz-Harrer-Str. 79, A-5020 Salzburg, Austria

**Keywords:** glioblastoma, epigenetic profiling, methylome, programmed cell death ligand 1, precision medicine

## Abstract

**Simple Summary:**

Glioblastomas are highly malignant brain tumors. Despite intensive research, there are no curative therapies available at the present time. Since Programmed Cell Death Ligand 1 (PD-L1) is a promising novel candidate in precision medicine, we here performed molecular analysis on glioblastomas with and without noteworthy PD-L1 expression. We found that there are severe molecular differences in glioblastomas depending on the PD-L1 state. An analysis of the top differences revealed post-transcriptional and RNA-associated pathways being altered. Targeting these altered pathways opens novel therapeutic approaches in the fight against brain cancer.

**Abstract:**

Glioblastomas are the most frequent primary brain tumors in adults. They show highly malignant behavior and devastating outcomes. Since there are still no targeted therapies available, median survival remains in the range of 12 to 15 months for glioblastoma patients. Programmed Cell Death Ligand 1 (PD-L1) is a promising novel candidate in precision medicine. Here, we performed integrated epigenome-wide methylation profiling of 866,895 methylation-specific sites in 20 glioblastoma samples comparing PD-L1 high- (i.e., TPS (tumor proportion score) > 30%) and PD-L1 low-expressing glioblastomas (i.e., TPS < 10%). We found 12,597 significantly differentially methylated CpGs (DMCG) (Δβ ≥ 0.1 and *p*-value < 0.05) in PD-L1 high- compared with PD-L1 low-expressing glioblastomas. These DMCGs were annotated to 2546 tiling regions, 139 promoters, 107 genes, and 107 CpG islands. PD-L1 high-expressing glioblastomas showed hypomethylation in 68% of all DMCGs. Interestingly, the list of the top 100 significantly differentially methylated genes showed the enrichment of regulatory RNAs with 19 DMCGs in miRNA, snoRNAs, lincRNAs, and asRNAs. Gene Ontology analysis showed the enrichment of post-transcriptional and RNA-associated pathways in the hypermethylated gene regions. In summary, dissecting the methylomes depending on PD-L1 status revealed significant alterations in RNA regulation and novel molecular targets in glioblastomas.

## 1. Introduction

Glioblastomas are the most malignant brain tumors in adults [1]. Thus, the World Health Organization (WHO, Geneva, Switzerland), which classifies central nervous system (CNS) tumors from CNS WHO Grade 1 to 4 based on malignant behavior, assigns glioblastomas as CNS WHO Grade 4 [1]. With three to four reported cases per 100,000 population in the western world, glioblastomas are also the most frequently diagnosed primary brain tumor in adult patients [1].

The current 2021 WHO classification for CNS tumors combines morphology and molecular information in an integrated diagnosis and a structured report [1]. Based on the molecular profile, gliomas are classified as astrocytomas, oligodendrogliomas, and glioblastomas [1]. Mutations in the isocitrate dehydrogenase (IDH) 1 and 2 genes are essential requirements for the diagnosis of astrocytoma, combined IDH1 and IDH2 mutations in combination with the complete loss of chromosomes 1p and 19q (loss of heterozygosity, LOH 1p/19q) are requirements for the diagnosis of oligodendroglioma, and *IDH1/2* as well as *H3F3A* (Histone H3 Family 3A), *HIST1H3B/C* wildtype status in combination with *TERT* (telomerase reverse transcriptase) mutations or *EGFR* (epithelial growth factor receptor) amplifications are requirements for the diagnosis of glioblastoma [1]. 

While astrocytomas and oligodendrogliomas are then assigned to CNS WHO Grades 2 to 4 for astrocytomas and 2 to 3 for oligodendrogliomas depending on distinct histological features, e.g., mitoses, microvascular proliferation, and necrosis, glioblastomas are classified as CNS WHO Grade 4 [1]. 

It is of note that there is a fundamental change in nomenclature of gliomas in the 2021 CNS WHO classification [1]. While, in previous classifications, Grade 4 gliomas were assigned to glioblastomas with or without *IDH* mutation, this has now changed. While the term glioblastoma is only used for *IDH* wildtype gliomas and no longer for *IDH*-mutated gliomas, the term astrocytoma Grade 4 is newly introduced for *IDH*-mutated gliomas with features of high malignant behavior (i.e., mitoses, microvascular proliferation, and necrosis) without 1p/19q loss. The differentiation between *IDH* wildtype glioblastoma WHO Grade 4 and *IDH*-mutated astrocytoma WHO Grade 4 now reflects the different underlying biology of these tumors.

With regard to molecular targets, gliomas frequently show methylation of the O6-methylguanin-DNA-methyltransferase (MGMT) promoter [2,3,4]. Hypermethylation of the MGMT promoter has been found to be associated with significantly longer survival in patients receiving an adjuvant radio-chemotherapy with temozolomide, according to the EORTC/NCIC protocol [5]. However, there has been no progress in glioma therapy to date.

A promising novel approach in glioma therapy is programmed cell death ligand 1 (PD-L1). PD-L1 has been identified as a potential candidate in precision medicine, e.g., in lung and gastrointestinal cancer [6,7,8]. As a key player in modulating interactions between tumors and the immune system, it triggers the immune response in many human cancers [6,7,8]. Interaction of PD-1 (programmed cell death 1) with PD-L1 inhibits the immune response by the induction of interleukin 10 (IL-10) in monocytes [9]. Thereby, an overexpression of PD-L1 as a druggable target [10,11,12,13,14,15] can be found in many tumors, e.g., lung, breast, and gastrointestinal cancer, and is associated with good response-applying PD-L1 inhibitors [16,17,18]. Indeed, PD-L1 overexpression has recently been detected in a subset of gliomas [19,20,21,22]. 

To better understand the molecular mechanisms occurring in glioblastoma IDH wildtype CNS WHO Grade 4 with and without PD-L1 expression, we performed epigenome-wide methylation analysis evaluating distinct molecularly altered pathways correlating with the PD-L1 expression status.

## 2. Materials and Methods

### 2.1. Tissue Collection and Immunohistochemical Analysis

For this study, we screened the archived samples of the University Institute of Pathology, University Hospital Salzburg, Paracelsus Medical University, collected between 2018 and 2021. The inclusion criteria for this study were defined as follows: a TPS PD-L1 score of >30% or <10%; and complete molecular profiling according to the 2021 CNS WHO classification; methylome profiling with the molecular glioblastoma subtypes mesenchymal, RTK I, RTK II, and midline. This resulted in the 20 samples to be analyzed. Details of the samples can be found in Supplementary Table S1.

All samples analyzed were formalin-fixed and paraffin-embedded (FFPE). Samples were reclassified according to the current 2021 WHO classification of CNS tumors. For this study, we selected samples that were allocated to the group of glioblastoma IDH wildtype CNS WHO Grade 4 based on molecular analysis [1]. Routine immunohistochemistry of the glioblastoma samples was performed on a Ventana BenchMark Ultra device (Roche, Grenzach-Wyhlen, BW, Germany) according to the manufacturer’s protocols for routine diagnostics applying ready to use antibodies against GFAP (760–4345) and Ki67 (790–4286). These markers were used only for routine diagnostics; there was no significant difference of GFAP and Ki67 expression in the two PD-L1 groups (Supplementary Table S1 and Supplementary Figure S1). PD-L1 expression was assessed by applying the PD-L1 22C3 antibody (M3653 kit antibody, Dako/Agilent, Santa Clara, CA, USA), and [19]; PD-L1 levels were quantified using the tumor proportion score (TPS) [23,24,25]. To determine the TPS, we counted the tumor cells with membranous positive staining and determined the fraction of PD-L1-positive tumor cells. Cases were considered adequate for inclusion if at least 100 tumor cells were present [23,24,25]. For this study, we selected 20 samples, 10 with PD-L1 TPSs of more than 30% (PD-L1 high group) and 10 with PD-L1 TPSs of less than 10% (PD-L1 low group). Based on our previous work [19], we found that 90% of gliomas showed PD-L1 TPSs in that range: 38% of gliomas showed PD-L1 TPS high expression, and 52% showed TPS low expression [19]. To reduce bias in TPS determination due to inter-tumoral variation in tissue and staining quality, we clearly separated the two groups of PD-L1 high and low expression, excluding samples with PD-L1 TPSs between 10% and 30%.

### 2.2. Molecular Genetic Analysis

Molecular genetic analyses for *IDH1*, *IDH2* mutations, as well as DNA methylation were performed as previously described [26,27,28]. In brief, representative tumor regions were microscopically identified and dissected. The DNA extraction was performed using the Maxwell system (Promega, Fitchburg, WI, USA) according to the manufacturer’s protocols. 

Mutational analysis of *IDH1* and *IDH2* genes was performed with either the AmpliSeq for Illumina Cancer Hotspot Panel v2 (Illumina, San Diego, CA, USA) or the AmpliSeq for Illumina Focus Panel (Illumina), respectively. Next generation sequencing was performed on an Illumina MiniSeq device following the manufacturer’s protocol [26,27,28].

Telomerase reverse transcriptase mutation analysis and MGMT methylation analysis were performed as described previously [26,27,28].

Methylation analysis was performed using the Infinium Methylation EPIC Bead Chip (Illumina) protocol [26,27,28]. Raw data files (idat-files) were analyzed using the molecularneuropathology.org bioinformatics pipeline of the German Cancer Research Center (DKFZ) and the v11b4 brain tumor classifier [29]. Molecular glioblastoma subfamilies were assessed using the molecular-neuropathology.org pipeline and the v11b4 brain tumor classifier [29].

Epithelial growth factor receptor amplification and LOH of chromosome 7 was assessed by CNV analysis of methylation profiles [29].

There was no significant difference on *TERT* mutations, MGMT methylation, *EGFR* amplification and LOH of chromosome 7 as well as molecular glioblastoma subfamilies (Supplementary Table S1 and Supplementary Figure S1).

### 2.3. Computational Data Analysis

Methylation data were processed using the Illumina Genome Studio Methylation Module and RnBeads [30]. Mapping was performed to the hg38 assembly. Methylation calls were transferred to beta values ranging from 0 to 1, i.e., 0% methylation to 100% methylation. Preprocessing included removal of SNP-enriched probes and Greycut filtering (i.e., filtering probes with the highest fraction of unreliable measurements one at a time in an iterative algorithm), context-specific probes, and probes on sex chromosomes [30]. For normalization, the Dasen method was utilized [30]. Probes were annotated with four genomic regions: tiling (i.e., genome tiling regions of length 5000), genes (version Ensemble genes 75), promoters (version Ensemble genes 75), and CpG islands (CpG island track of the UCSC Genome Browser). Computation of *p*-values on site level was performed using the Limma method (i.e., hierarchical linear model fitted using an empirical Bayes approach). As differential method, we selected diffVar. We used nominal *p*-values as performed in other epigenome-wide association studies (EWAS) [31,32]. Further analyses were performed using Morpheus and GraphPad Prism 9 software suite.

## 3. Results

### 3.1. Glioblastoma IDH Wildtype CNS WHO Grade 4 Show Different Amount of PD-L1 Expression Quantified by Tumor Proportion Score (TPS)

**Figure 1:**
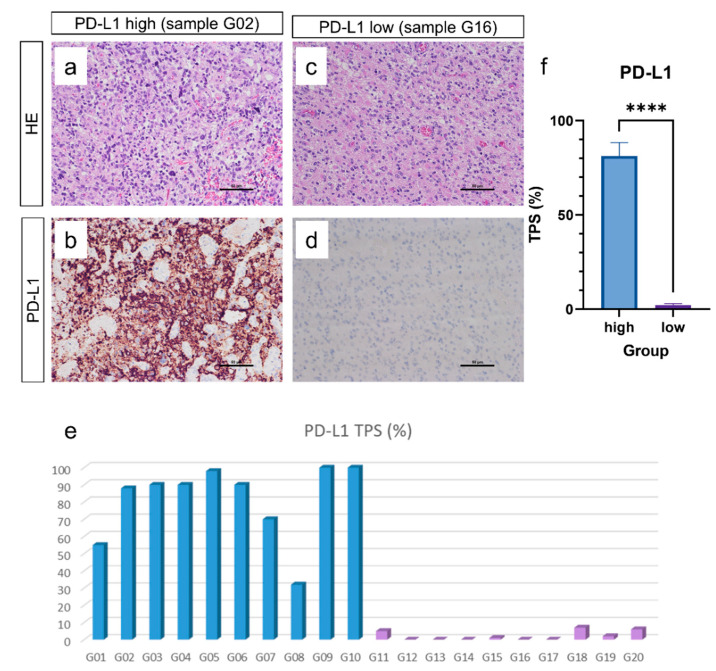
Analysis of PD-L1 expression. Analysis of PD-L1 expression in glioblastoma IDH wildtype CNS WHO Grade 4 was performed applying tumor proportion score (TPS). Twenty glioblastoma samples were included with ten samples showing PD-L1 high expression (i.e., more than 30%) (**a**,**b**) and ten showing PD-L1 low expression (i.e., less than 10%) (**c**,**d**) with mean of TPS 81.3% in the PD-L1 high group and mean of TPS 2.1% in the PD-L1 low group (**e**,**f**). (**a**–**d**): scale bar: 50 µm; f: group size: PD-L1 high: 10 samples, PD-L1 low: 10 samples. **** *p* < 0.0001.

In this study, we analyzed 20 glioblastoma samples. The inclusion criteria for this study were defined as follows: TPS PD-L1 score of >30% or <10%; complete molecular profiling according to the 2021 CNS WHO classification; methylome profiling with the molecular glioblastoma subtypes mesenchymal, RTK I, RTK II, and midline. This resulted in 20 samples to be analyzed: 10 samples with TPS PD-L1 scores of less than 10%, and 10 samples with TPS PD-L1 scores of more than 30%. To reduce bias in TPS determination due to inter-tumoral variation in tissue and staining quality, no samples with TPS PD-L1 score between 10% and 30% were included. Details on samples can be found in Supplementary Table S1. The resulting mean PD-L1 TPS was 81.3% in the high group and 2.1% in the low group (Figure 1a–f). 

### 3.2. Differential Methylation Analysis Shows PD-L1 Correlation with Methylation Signatures in Glioblastoma IDH Mutant CNS WHO Grade 4

**Figure 2:**
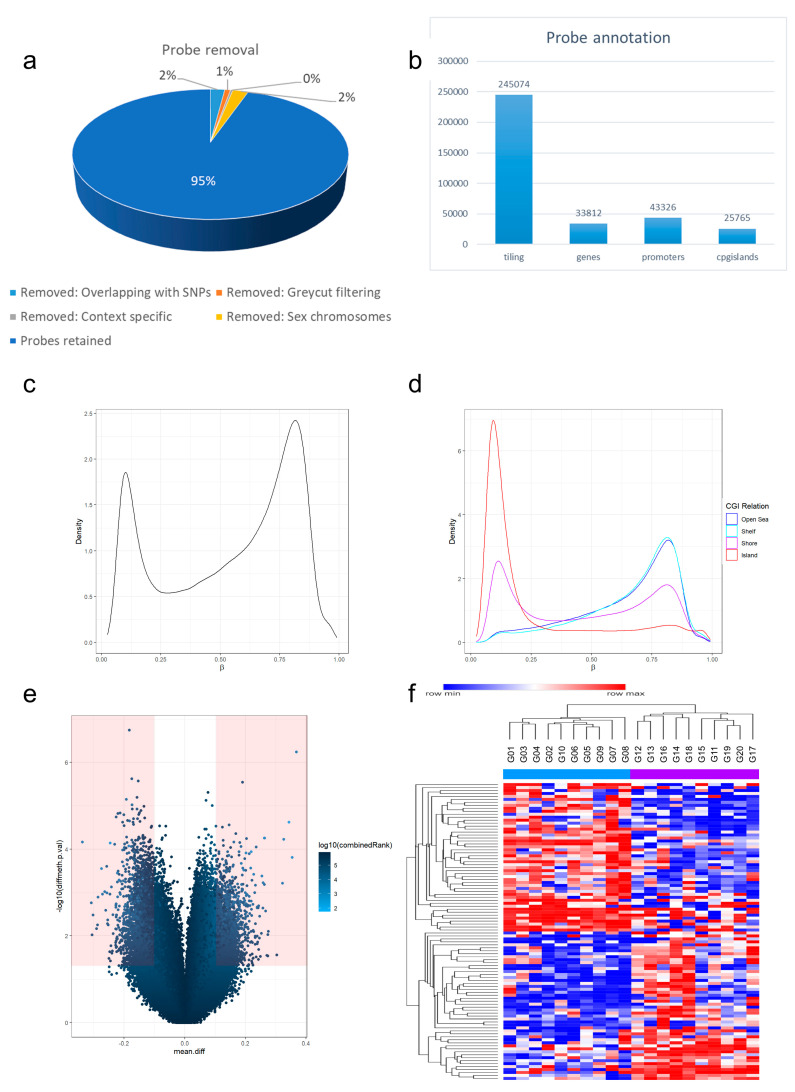
Exploratory methylation analysis. After probe removal and filtering, exploratory methylation analysis was performed on 820,848 probes (**a**). These retained probes were annotated with genomic region tiling, genes, promoters, and CpG islands (**b**). Analysis of global methylation distribution showed uneven distribution (**c**). Analysis of probe categories showed high densities of unmethylated values in CpG islands while high densities of methylated values were found in open sea regions (**d**). Volcano plot shows pairwise methylation analysis resulting in 12,597 significantly differentially methylated probes with Δβ values of ≥0.1 and *p*-values of <0.05 that are indicated in red (**e**). Considering the 100 most variable CpGs, we were able to dissect the glioblastomas with high and low PD-L1 expression (**f**).

Applying the Illumina Infinium EPIC bead chip, we interrogated 866,895 methylation-sensitive probes per sample. During preprocessing, we removed 17,371 probes that overlapped with SNPs, 6915 probes applying greycut filtering, 2940 context-specific probes, and 18,821 probes on sex-chromosomes; this resulted in a total of 820,848 retained probes (Figure 2a). These were annotated with genomic region tiling (245,074 probes), genes (33,812 probes), promoters (43,326), and CpG islands (25,765) (Figure 2b). An analysis of global beta value distributions showed high densities with beta values <0.1 and >0.8 (Figure 2c). Annotating methylation sites with probe categories showed that CpG islands showed high densities of unmethylated beta values while shelf and open sea regions showed higher densities of methylated values with an intermediate distribution of shores (Figure 2d). Differential methylation analysis was performed with regard to PD-L1 status. As a mathematical approach to identify significantly differentially methylated sites, we performed pairwise comparisons resulting in 12,597 significantly differentially methylated probes with Δβ values of ≥0.1 and *p*-values of <0.05. In the generated volcano plot, these significantly differentially methylated probes are indicated in red (Figure 2e). The dots on the upper left and right side are significantly hyper- (right upper side) or hypo-methylated (upper left side) in PD-L1 high- versus low-expressing glioblastomas. 

Since the mathematical approach was a differential analysis, the two groups of PD-L1-expressing glioblastomas were compared: PD-L1 high versus low. 

For PD-L1 high-expressing glioblastomas, the dots on the upper right side represent CGs that are significantly hyper-methylated in PD-L1 high versus PD-L1 low glioblastomas. The dots on the upper left side represent CGs that are hypo-methylated in PD-L1 high- versus PD-L1 low-expressing glioblastomas. 

In terms of PD-L1 low-expressing glioblastomas, the results are vice versa: the dots on the upper right side represent CGs that are significantly hypo-methylated in PD-L1 low versus PD-L1 high glioblastomas. The dots on the upper left side represent CGs that are hyper-methylated in PD-L1 low- versus PD-L1 high-expressing glioblastomas.

Next, we performed hierarchical clustering of differentially methylated CpGs (DMCG). Considering the 100 most variable CpGs, the two groups of PD-L1 TPS high- and low-expressing glioblastomas showed clustering using Manhattan distance (Figure 2f). This approach enables the identification of DMCG denominators of the glioblastomas reflecting the PD-L1 status.

### 3.3. Enrichment Analysis of DMCGs Reveals Distinct Altered Pathways Correlating with PD-L1 Status 

**Figure 3:**
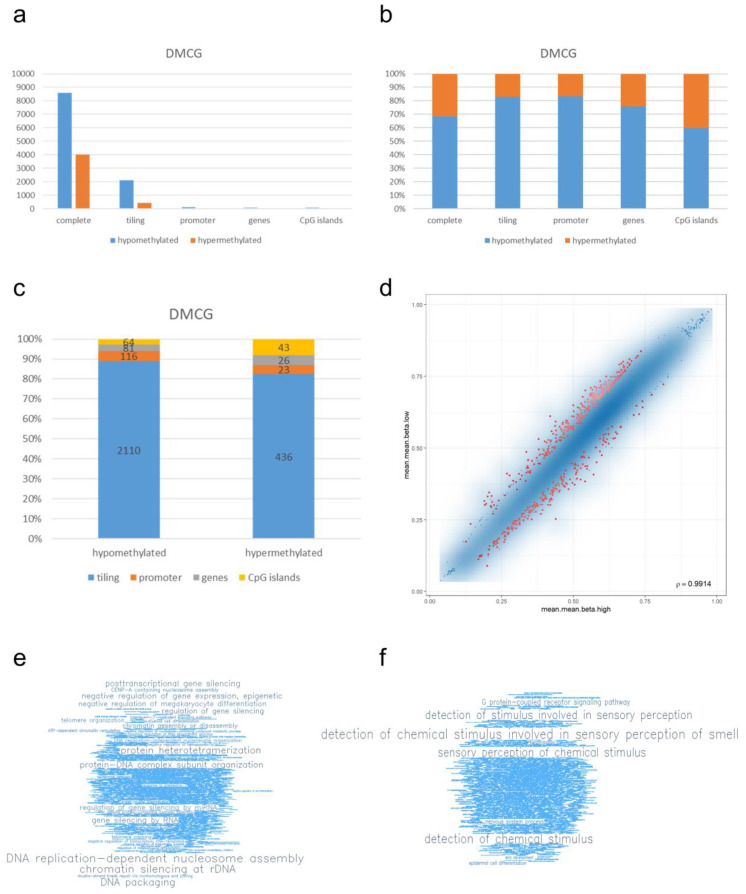
Differential methylation analysis. Differential methylation analysis revealed 12,597 DMCGs (**a**). Thereby, PD-L1 high-expressing glioblastomas showed global hypomethylation compared with PD-L1 low-expressing glioblastomas (**b**). Allocating DMCGs on genomic positions revealed more than 80% being located in tiling regions while less than 10% are in promoter, gene and CpG island regions (**c**). For GO enrichment analysis, top 500 ranked regions (indicated in red) were processed (**d**). Word clouds show GO term enrichments of “DNA replication-dependent nucleosome assembly”, “chromatin silencing at rDNA”, and “DNA packaging” in hypermethylated gene regions, and (**e**) “G protein-coupled receptor signaling pathway” in hypomethylated gene regions (**f**).

Next, we performed enrichment analysis of the identified DMCGs. The 12,597 significantly differentially methylated CpG sites (DMCG) can be annotated with 2546 tiling regions, 139 promoter regions, 107 genes, and 107 CpG islands (Figure 3a). 

Interestingly, glioblastomas with high PD-L1 expression showed global hypomethylation in differentially methylated probes, i.e., 68% hypomethylation of all differentially methylated probes, 83% of tiling regions, 83% of promoter regions, 76% of genes, and 60% of CpG islands (Figure 3b). 

An analysis of DMCGs and genomic position showed that more than 80% of DMGCs are in tiling regions while less than 10% are in promoter, gene, and CpG island regions (Figure 3c). Thereby, hypo-/and hypermethylated CGs are identified by comparing the group of PD-L1 high- versus PD-L1 low-expressing glioblastomas (Figure 3c and Supplementary Figure S2). According to the underlying mathematical model, hyper-methylated CGs show a higher degree of methylation in PD-L1 high glioblastomas versus PD-L1 low glioblastomas. Hypo-methylated CGs show a lower degree of methylation in PD-L1 high glioblastomas versus PD-L1 low glioblastomas. In terms of PD-L1 low-expressing glioblastomas, the results are vice versa. 

As exemplarily examples of differentially methylated genes, we picked genes from the top 100 list due to (1) the position in the top DMCGs, (2) known strong tumor association, and/or (3) family members of genes known to be biologically relevant in gliomas. Of note, the genes *HNRNPH1* (heterogeneous nuclear ribonucleoprotein H1) as top two, *HOXA9* (homebox) as top seven, and histone variant *HIST1H2BM* as top thirty-seven, were the top matches. Interestingly, of the 100 top DMCGs there were nineteen allocated with regulatory RNAs: six miRNAs (miR196B (top one) (promoter and gene body), miR550A1, miR4708, and miR2267), two small nucleolar RNAs (SNORD114-31, SNORA15), five long non-coding RNAs (LINC00499, LINC01036, LINC00989, LINC00474, and LINC00654) and seven anti-strand RNAs (IL21R-AS1, ISM1-AS1, DLX6-AS2, LATS2-AS1, TBX5-AS1, HOXA10-AS, and DLX6-AS2) (Supplementary Table S2).

In order to perform Gene Ontology (GO) enrichment analysis, a region rank was calculated following three criteria: the differences in mean methylation, the quotient of mean methylation, and the statistical testing. The smaller this combined region rank is, the more evidence for differential methylation is exhibited. The scatter plot indicates the top 500 ranked regions (Figure 3d). Gene ontology (GO) enrichment analysis of the top 500 ranked differentially methylated proteins showed GO term enrichments of pathways being associated with post-transcriptional and RNA-associated gene regulation: GO analysis showed “DNA replication-dependent nucleosome assembly”, “chromatin silencing at rDNA”, “DNA packaging”, “posttranscriptional gene silencing”, “negative regulation of gene expression, epigenetic”, “gene silencing by RNA”, “regulation of gene silencing”, “regulation of gene silencing by mRNA”, and “gene silencing by miRNA” among the top 45 hypermethylated pathways in the PD-L1 high samples (Figure 3e, Supplementary Table S3). Among the top 45 hypomethylated pathways in the PD-L1 high samples, there were pathways being associated with “posttranscriptional gene silencing by RNA”, “T-cell activation involved in immune response”, “leukocyte mediated cytotoxicity”, and “cell killing” (Figure 3f, Supplementary Table S4).

## 4. Discussion

PD-L1 is a key player in personalized anti-tumor therapy [10,11,12,13,14,15]. Thereby, PD-L1 is expressed on the cell surface of many tumors, e.g., lung, urothelial, and breast [16,17,18], and is an efficient target of PD-L1 inhibitors such as pembrolizumab in a clinical setting [33]. 

PD-L1 effects immune escape by tumor immunity [21]. Thereby, PD-L1 interacts with PD-1 (programmed cell death 1), inhibiting the immune response that is induced by interleukin 10 [9]. Thereby, the PD-1/PD-L1 pathway restrains the hyperactivation of the immune cells and is a feasible approach to regulate the tumor microenvironment in cancers [34]. Survival analysis that was performed by [21] showed that there is no survival difference in glioblastomas with high versus low PD-L1 expression (without pembrolizumab therapy). Thus, there are further confounding factors that are associated in the complex network of PD-L1 and miR196B expression and patient survival. We added this aspect in the discussion. Studies that examine the therapeutic benefit of PD-L1 expression in glioblastomas and pembrolizumab therapy are currently ongoing [35]. They have found that patients with high PD-L1 expression may benefit from pembrolizumab therapy. 

In a previous study, we demonstrated that PD-L1 is also expressed in a subfraction of glioblastomas [19]. Based on these findings, we now performed epigenome-wide methylation analysis of glioblastomas with high/low PD-L1 expression to reveal differentially methylated genes and pathways associated with PD-L1 expression to better understand the underlying biological processes. 

We found that there are distinct significantly differentially methylated regions in glioblastomas correlating with the PD-L1 expression status: 12,597 CpGs showed methylation differences of ≥10% with *p*-values of <0.05. Of these CpGs, 2546 can be assigned to genome tiling regions, i.e., genomic regions of a length of 5000, 139 can be assigned to promoter regions, 107 can be assigned to genes, and 107 can be assigned to CpG islands. Interestingly, there was a larger amount of global (i.e., all analyzed methylation sites) and local (i.e., assigned to distinct genomic regions) hypomethylation in glioblastomas with PD-L1 high expression (methylation differences of ≥10% with *p*-values of <0.05): 68% (8593 of 12,597) of all significantly different methylation sites were hypomethylated in PD-L1 high glioblastomas, 83% of tiling region CpGs (2110 of 2546), 83% of all promoter-associated CpGs (116 of 139), 76% of CpGs associated with genes (81 of 107) and 60% of CpGs associated with CpG islands (64 of 107). Since hypomethylation is associated with the activation of transcription, these findings suggest a predominant gene activation in PD-L1 high-expressing glioblastomas. 

Interestingly, the list of the top 100 significant differentially methylated genes contained tumor-associated genes *HNRNPH1* (top two), *HOXA9* (top seven) and *HIST1H2BM* (top thirty-seven) among the top matched. *HNRNPH1* acts as a regulator of cell proliferation and progression of disease in chronic myeloid leukemia [36,37], *HOXA9* acts as an anti-apoptotic gene promoting MYC-mediated leukemogenesis by the ability to maintain gene expression for multiple anti-apoptotic pathways [38,39], and *HIST1H2BM* is known to be expressed in breast cancer [40,41]. 

Furthermore, miRNAs (miR196B, miR550A1, miR4708, and miR2267) [42,43], small nucleolar RNAs (SNORD114-31, SNORA15) [44,45], and long non-coding RNAs (LINC00499, LINC01036, LINC00989, LINC00474, and LINC00654) [46,47] were found among the top matches. As the top differentially methylated gene, miR196B was significantly hypermethylated in PD-L1 high-expressing glioblastoma. This is well in line with published reports showing that the high expression of miR196B is associated with a poor prognosis in patients with ovarian cancer [42]. 

In glioblastomas, Ma et al. examined miR196B expression [48]. They found that upregulation of miR196B in glioblastomas is associated with a poor prognosis. This phenomenon is analogous to ovarian cancer [42]. Here, we found that miR196B is the top one hit of differentially methylated genes (Supplementary Table S2). In PD-L1 high-expressing glioblastomas, miR196B is hypermethylated compared with PD-L1 low-expressing glioblastomas. An interesting aspect would be the significance of PD-L1 expression on patient outcome in glioblastomas. Due to the limited number of cases analyzed, there was no survival analysis performed. Survival analysis performed by Heiland et al., 2017, showed that there is no survival difference in glioblastomas with high versus low PD-L1 expression (without pembrolizumab therapy) [21]. Thus, there are further confounding factors that are associated in the complex network of PD-L1 and miR196B expression and patient survival.

Gene ontology enrichment analysis revealed that differential methylated sites are associated with the pathways of “DNA replication-dependent nucleosome assembly”, “chromatin silencing at rDNA”, and “DNA packaging” in hypermethylated gene regions. These findings show the close association of the PD-L1 expression status with epigenomic interactions with the tumor microenvironment, immune response, and cellular proliferation.

There are already ongoing studies that examine the therapeutic benefits of PD-L1 expression in glioblastomas and pembrolizumab therapy [35]. They have found that patients with high PD-L1 expression may benefit from pembrolizumab therapy. Further approaches may target, e.g., identified miRNAs such as miR196B. In glioblastoma therapy, there is the general problem of the blood–brain barrier. Small molecules targeting these novel identified RNAs and genes may be better in passing the blood–brain barrier. Thus, further studies may be necessary to reveal clinical benefits.

PD-L1 interacts in the complex network of the tumor microenvironment. In the context of cancer, the normal regulation of the PD-1/PD-L1-pathway is disrupted, enabling tumor cells to escape immune surveillance [34]. An analysis of the PD-L1 promoter itself did not show significant differences in promoter methylation (Supplementary Figure S2). Therefore, the activation of PD-L1 in glioblastoma is not a consequence of the hypermethylation profile. With regard to the top DMCGs, it is of high interest that there are numerous DMGCs that are associated with post-transcriptional and RNA-associated gene regulation. This may suggest that PD-L1 expression is rather a cause than a consequence of the hypermethylation profile. However, further studies are necessary to answer this question.

In summary, we were able to dissect differential methylated pathways in glioblastomas correlating with PD-L1 TPS expression, thereby identifying potential novel molecular targets for future precision medicine. There are already studies ongoing that examine the therapeutic benefit of PD-L1 expression in glioblastomas and pembrolizumab therapy [35]. They have found that patients with high PD-L1 expression may benefit from pembrolizumab therapy. Further approaches may target e.g., identified miRNAs, such as miR196B. In glioblastoma therapy, there is the general problem of the blood–brain barrier. Small molecules targeting these novel identified RNAs and genes may be better at passing the blood–brain barrier. Thus, further studies may be necessary to reveal clinical benefits. 

## 5. Conclusions

Despite intensive research, glioblastomas still show highly malignant behavior and devastating outcomes with no curative therapy available. Since PD-L1 is a promising novel candidate in precision medicine, we here performed integrated epigenome-wide methylation profiling comparing PD-L1 high- and low-expressing glioblastomas. Applying this approach, we identified distinct DMCGs depending on the PD-L1 expression state. Interestingly, the top 100 DMCGs showed an overrepresentation of regulatory RNAs and GO analysis showed enrichment of post-transcriptional and RNA-associated pathways in hypermethylated gene regions. Targeting these altered pathways opens novel therapeutic targets in the fight against glioblastoma.

## Data Availability

Data are available from the corresponding author on request.

## Supplementary Materials

The following supporting information can be downloaded at: https://www.mdpi.com/article/10.3390/cancers14215375/s1, Figure S1: Analysis of immunohistochemical and molecular hallmarks of PD-L1 high- and low-expressing glioblastomas.; Figure S2: Analysis of PD-L1 high and low glioblastomas. Table S1: Detailed characteristics of all 20 samples; Table S2: Top 100 significant differentially methylated genes.; Table S3: GO enrichment analysis of top 45 hypermethylated pathways in PD-L1 high-expressing glioblastomas; Table S4: GO enrichment analysis of top 45 hypomethylated pathways in PD-L1 high-expressing glioblastomas.
