# Supplementary material for: Methylome Profiling of PD-L1-Expressing Glioblastomas Shows Enrichment of Post-Transcriptional and RNA-Associated Gene Regulation
Source: Cancers (Basel). 2022 Oct 31;14(21):5375. doi: 10.3390/cancers14215375 (PMC9656473; doi:10.3390/cancers14215375)
Supplement: Supplementary file 1 [file cancers-14-05375-s001.zip › cancers-1935994-supplementary.pdf]

Supplementary material:

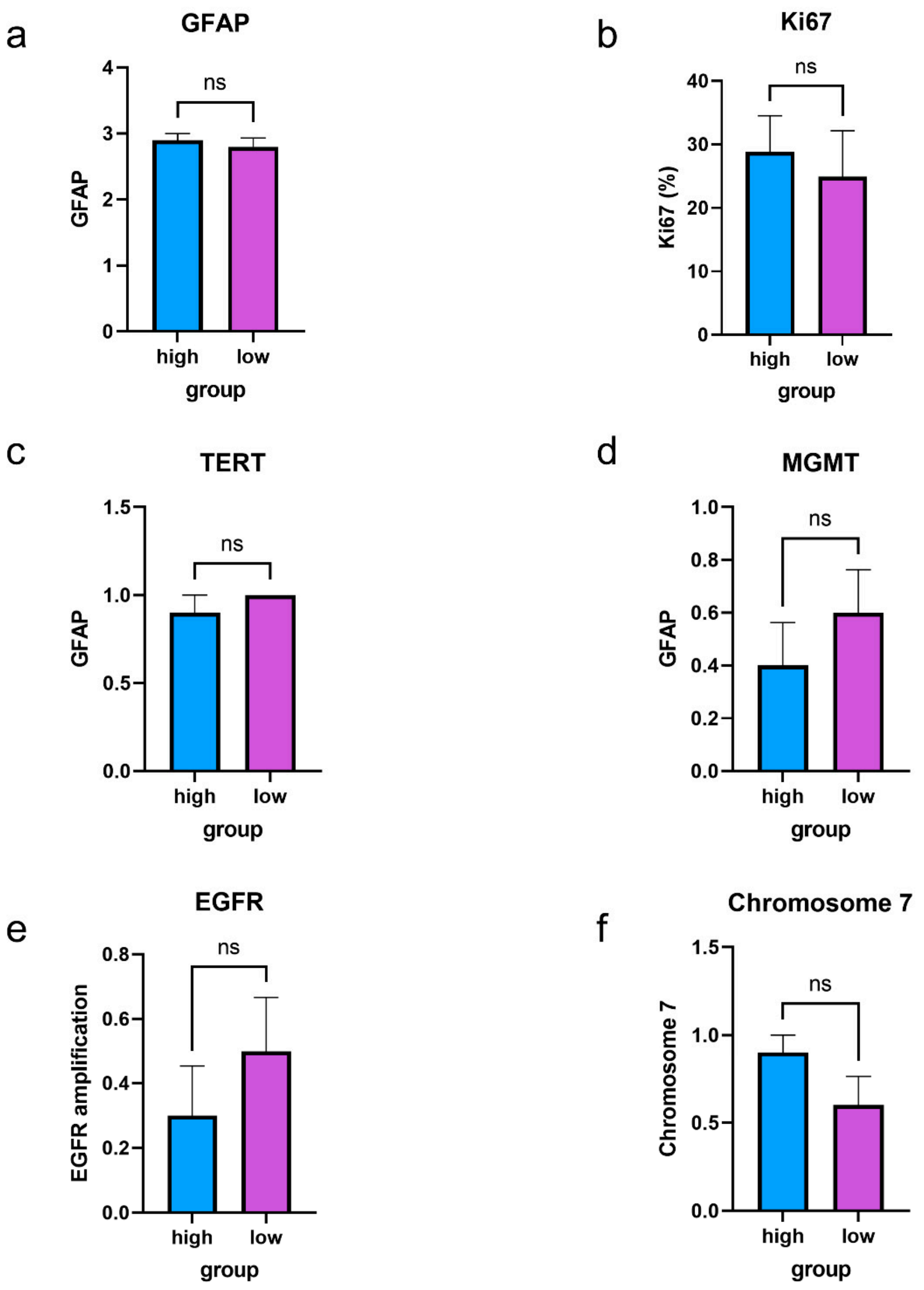

Supplementary Figure S1. Analysis of immunohistochemical and molecular hallmarks of PD-L1 high and low expressing glioblastomas. There was no significant difference in GFAP (a) and Ki67 (b) expression, TERT mutations (c) and MGMT methylation (d), EGFR amplification (e) and LOH of Chromosome 7 (f). (a): Expression scale: 0: no expression, 3: very high expression; (b) fraction per 100 tumor cells; (c) 0: no mutation, 1: mutation; (d) 0: no methylation, 1: methylation; (e) 0: no amplification, 1: amplification; (f) 0: no LOH Chromosome 7, 1: LOH Chromosome 7. ns: not significant.

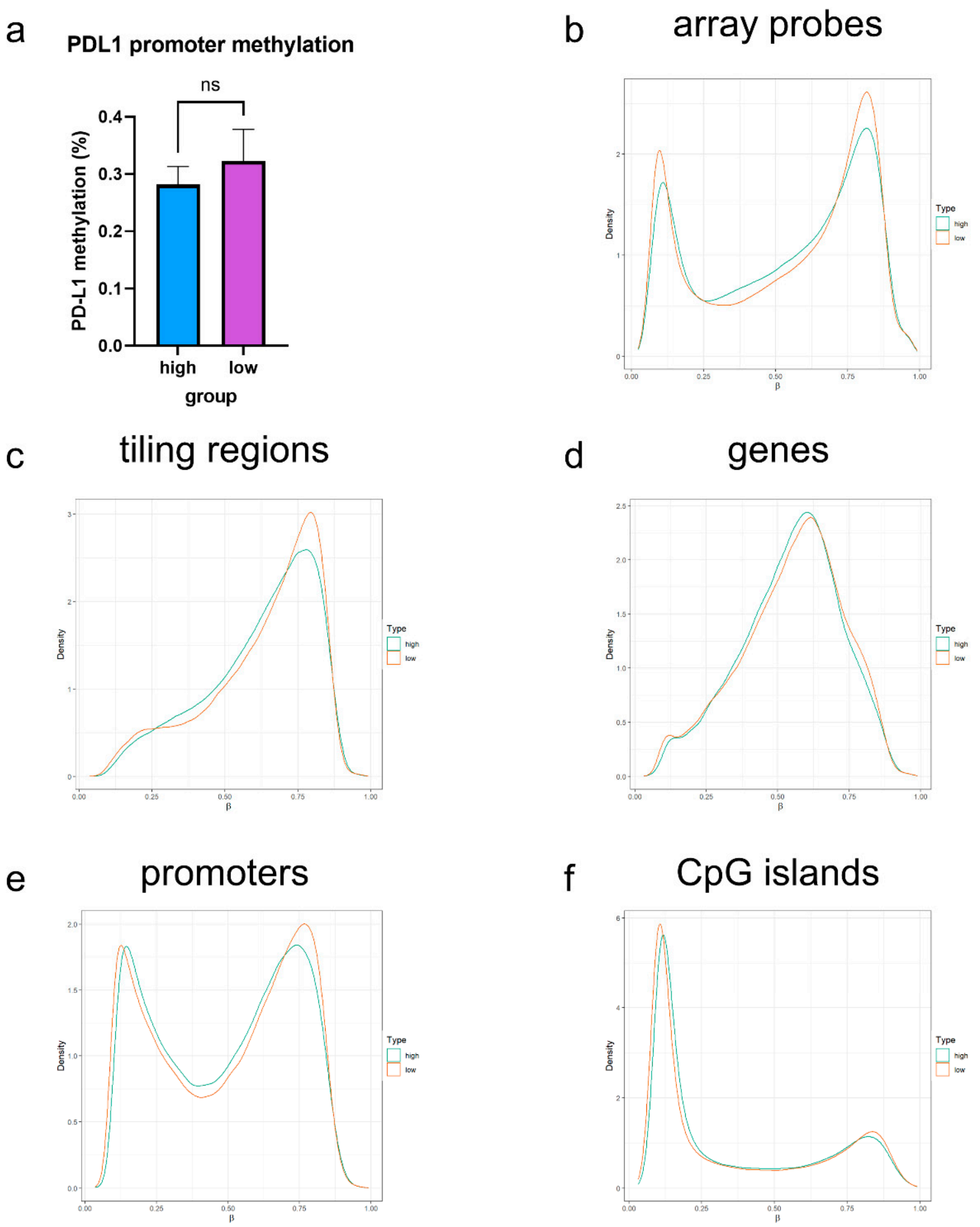

Supplementary Figure S2. Analysis of PD-L1 high and low glioblastomas. (a) PD-L1 promoter methylation did not show significant differences in the analyzed groups. Comparing the distributions of methylation values (β values) in the two PD-L1 groups defined by array probes (b), tiling regions (c), genes (d), promoters (e) and CpG islandes (f). Differences (DMCGs) applying differential methylation analysis can be found in Figure 3. ns: not significant.

Supplementary Table S1. Detailed characteristics on all 20 samples. Indicated are age, sex, PD-L1 TPS, PD-L1 promoter methylation (cg19724470), EGFR amplification status, LOH of Chromosome 7, GFAP and Ki67 staining as well as TERT mutation, MGMT methylation status and molecular subfamily. GFAP: Expression scale: 0: no expression, 3: very high expression; Ki67 fraction per 100 tumor cells; TERT 0: no mutation, 1: mutation; MGMT 0: no methylation, 1: methylation; EGFR 0: no amplification, 1: amplification; Chromosome 7 0: no LOH Chromosome 7, 1: LOH Chromosome 7.

Sample ID	Age (y)	Sex	Diagnosis (5. Edition CNS WHO Classification)	IDH1	IDH2	PD-L1 Group	PD-L1 Status	PDL1_TPS (%)	PD-L1 promoter (%)	EGFR	LOH Chr 7	GFAP (0-3+)	Ki67 (%)	TERT (wt=0, mut=1)	MGMT (u=0, m=1)	Methylationfamily (v11b4)	Subclass (v11b4)
G01	64	m	Glioblastoma IDH Wildtype CNS WHO Grade 4	wt	wt	PDL1_high_01	high	55	0.19	1	1	3	45	1	1	Glioblastoma IDH wt	RTK II
G02	32	m	Glioblastoma IDH Wildtype CNS WHO Grade 4	wt	wt	PDL1_high_02	high	88	0.25	0	1	3	22	1	0	Glioblastoma IDH wt	RTK II
G03	75	f	Glioblastoma IDH Wildtype CNS WHO Grade 4	wt	wt	PDL1_high_03	high	90	0.38	0	1	3	17	1	0	Glioblastoma IDH wt	RTK II
G04	77	f	Glioblastoma IDH Wildtype CNS WHO Grade 4	wt	wt	PDL1_high_04	high	90	0.27	1	1	3	18	1	0	Glioblastoma IDH wt	RTK II
G05	66	m	Glioblastoma IDH Wildtype CNS WHO Grade 4	wt	wt	PDL1_high_05	high	98	0.32	0	0	3	35	1	1	Glioblastoma IDH wt	mesenchymal
G06	59	m	Glioblastoma IDH Wildtype CNS WHO Grade 4	wt	wt	PDL1_high_06	high	90	0.29	1	1	3	35	1	0	Glioblastoma IDH wt	RTK II
G07	45	f	Glioblastoma IDH Wildtype CNS WHO Grade 4	wt	wt	PDL1_high_07	high	70	0.49	0	1	3	20	1	1	Glioblastoma IDH wt	RTK I
G08	25	m	Glioblastoma IDH Wildtype CNS WHO Grade 4	wt	wt	PDL1_high_08	high	32	0.28	0	1	3	70	0	0	Glioblastoma IDH wt	midline
G09	52	f	Glioblastoma IDH Wildtype CNS WHO Grade 4	wt	wt	PDL1_high_09	high	100	0.19	0	1	2	10	1	0	Glioblastoma IDH wt	mesenchymal
G10	65	f	Glioblastoma IDH Wildtype CNS WHO Grade 4	wt	wt	PDL1_high_10	high	100	0.16	0	1	3	16	1	1	Glioblastoma IDH wt	mesenchymal
G11	55	m	Glioblastoma IDH Wildtype CNS WHO Grade 4	wt	wt	PDL1_low_01	low	5	0.17	0	1	2	3	1	0	Glioblastoma IDH wt	mesenchymal
G12	66	m	Glioblastoma IDH Wildtype CNS WHO Grade 4	wt	wt	PDL1_low_02	low	0	0.28	1	0	3	3	1	1	Glioblastoma IDH wt	mesenchymal
G13	52	m	Glioblastoma IDH Wildtype CNS WHO Grade 4	wt	wt	PDL1_low_03	low	0	0.18	1	1	3	25	1	0	Glioblastoma IDH wt	RTK II

G14	62	m	Glioblastoma IDH Wildtype CNS WHO Grade 4	wt	wt	PDL1_low_04	low	0	0.55	0	1	3	40	1	1	Glioblastoma IDH wt	RTK II
G15	81	m	Glioblastoma IDH Wildtype CNS WHO Grade 4	wt	wt	PDL1_low_05	low	1	0.21	1	0	3	8	1	1	Glioblastoma IDH wt	mesenchymal
G16	71	m	Glioblastoma IDH Wildtype CNS WHO Grade 4	wt	wt	PDL1_low_06	low	0	0.29	0	1	3	32	1	1	Glioblastoma IDH wt	RTK I
G17	47	m	Glioblastoma IDH Wildtype CNS WHO Grade 4	wt	wt	PDL1_low_07	low	0	0.34	1	1	3	25	1	0	Glioblastoma IDH wt	RTK II
G18	77	m	Glioblastoma IDH Wildtype CNS WHO Grade 4	wt	wt	PDL1_low_08	low	7	0.72	0	1	2	80	1	1	Glioblastoma IDH wt	RTK I
G19	74	m	Glioblastoma IDH Wildtype CNS WHO Grade 4	wt	wt	PDL1_low_09	low	2	0.25	1	0	3	13	1	1	Glioblastoma IDH wt	mesenchymal
G20	43	f	Glioblastoma IDH Wildtype CNS WHO Grade 4	wt	wt	PDL1_low_10	low	6	0.24	0	0	3	20	1	0	Glioblastoma IDH wt	mesenchymal

Supplementary Table S2. Top 100 significant differentially methylated genes. Analysis showed HNRNPH1, a regulator of cellular proliferation, HOXA9, an anti-apoptotic gene, as well as 19 DMCGs in regulatory RNAs, i.e. miRNA, snoRNAs, lincRNAs and asRNAs among top 100 matches.

id	Chromosome	Start	End	Symbol	Location	mean.mean.high	mean.mean.low	mean.mean.diff	mod.mean.mean.diff2	comb.p.val	combinedRank
ENSG00000207584	chr7	27209099	27209183	MIR196B	gene	0.438856551	0.252662052	0.186194499	0.186194499	0.001625113	9
ENSG00000169045	chr5	179061286	179063285	HNRNPH1	promoter	0.138656206	0.317495533	-0.178839327	0.178839327	0.006069463	73
ENSG00000256355	chr12	67818267	67820266	NTAN1P3	promoter	0.535292541	0.695121721	-0.15982918	0.15982918	0.003862197	241
ENSG00000222308	chr5	151254754	151256753	RNA5SP198	promoter	0.604680408	0.764014822	-0.159334414	0.159334414	0.003581832	377
ENSG00000258806	chr14	20696061	20698060	OR11H7	promoter	0.762662904	0.604631471	0.158031432	0.158031432	0.000946893	392
ENSG00000257569	chr12	56292584	56294583	GSTP1P1	promoter	0.602678813	0.760210014	-0.157531201	0.157531201	0.001338591	391
ENSG00000078399	chr7	27209618	27211617	HOXA9	promoter	0.556835423	0.401850837	0.154984586	0.154984586	0.008187104	101
ENSG00000145309	chr4	71200683	71202833	CABS1	gene	0.49976729	0.648854449	-0.149087159	0.149087159	0.007370938	152
ENSG00000232382	chr3	98188324	98189420	OR5K1	gene	0.560670915	0.70966431	-0.148993395	0.148993395	0.001057733	221
ENSG00000207584	chr7	27208684	27210683	MIR196B	promoter	0.458805106	0.314656303	0.144148803	0.144148803	0.009250607	113
ENSG00000259699	chr15	89679227	89681226	HMGB1P8	promoter	0.481896736	0.622110559	-0.140213823	0.140213823	0.020784131	303
ENSG00000228319	chr13	56788507	56791076	SPATA2P1	gene	0.436749016	0.576644234	-0.139895218	0.139895218	0.027713441	296
ENSG00000227425	chr1	157829487	157831486	MRPS21P2	promoter	0.716898967	0.856791703	-0.139892736	0.139892736	0.013305882	1024
ENSG00000227177	chr1	172854899	172856898	AIMP1P2	promoter	0.537273915	0.397610373	0.139663542	0.139663542	0.028654352	450
ENSG00000250339	chr4	74809728	74810041	CXCL1P	gene	0.206118607	0.345253534	-0.139134927	0.139134927	0.037166843	427
ENSG00000220586	chr6	39960554	39967972	TUBBP9	gene	0.594022141	0.732635438	-0.138613297	0.138613297	0.003573987	329
ENSG00000271956	chr7	96636523	96638522	DLX6-AS2	promoter	0.589478274	0.451346288	0.138131986	0.138131986	0.011210405	149
ENSG00000237393	chr10	47964260	47966259	SLC9A3P4	promoter	0.363409244	0.499932076	-0.136522832	0.136522832	0.04979276	1003
ENSG00000253187	chr7	27208238	27211534	HOXA10-AS	gene	0.52546746	0.390405586	0.135061874	0.135061874	0.014483122	136
ENSG00000180881	chr12	75784209	75786208	CAPS2	promoter	0.139774265	0.274526401	-0.134752136	0.134752136	0.005286701	59
ENSG00000206782	chr4	132192897	132194896	RNU6-224P	promoter	0.626830467	0.76118384	-0.134353373	0.134353373	0.002663278	774
ENSG00000171603	chr1	9884085	9886084	CLSTN1	promoter	0.212047971	0.344256332	-0.132208361	0.132208361	0.020747044	302
ENSG00000226666	chr2	222826456	222828484	HSPA9P1	gene	0.581730352	0.450733176	0.130997176	0.130997176	0.047194161	594
ENSG00000180481	chr12	75784850	75826468	GLIPR1L2	gene	0.201503091	0.331275376	-0.129772286	0.129772286	0.005774665	50
ENSG00000230426	chr1	187610358	187612992	ERVMER61-1	gene	0.581232743	0.709470154	-0.128237412	0.128237412	0.018914109	386
ENSG00000251372	chr4	139229365	139231364	LINC00499	promoter	0.653254643	0.781107412	-0.127852769	0.127852769	0.003710257	1016

ENSG00000255399	chr12	114845996	114850636	TBX5-AS1	gene	0.483598797	0.356383731	0.127215066	0.127215066	0.049836501	638
ENSG00000230671	chr4	119321669	119321989	NDUFS5P5	gene	0.277632766	0.404677403	-0.127044637	0.127044637	0.006503688	56
ENSG00000230671	chr4	119321490	119323489	NDUFS5P5	promoter	0.277632766	0.404677403	-0.127044637	0.127044637	0.006503688	77
ENSG00000243591	chr18	6600039	6602038	RN7SL282P	promoter	0.500202383	0.627161481	-0.126959098	0.126959098	0.023915082	434
ENSG00000197617	chr1	247419374	247420445	VN1R5	gene	0.501433001	0.627780834	-0.126347833	0.126347833	0.026614057	283
ENSG00000214313	chr7	99578385	99585158	AZGP1P1	gene	0.66798933	0.793998082	-0.126008751	0.126008751	0.005970115	623
ENSG00000265646	chr17	25410563	25412562	TUFMP1	promoter	0.632545802	0.757284601	-0.124738799	0.124738799	0.001663274	999
ENSG00000215545	chr20	29891015	29896388	DEFB116	gene	0.463679236	0.588248114	-0.124568879	0.124568879	0.007058576	215
ENSG00000229612	chr1	168865874	168867873	SUMO1P2	promoter	0.431561199	0.555683901	-0.124122702	0.124122702	0.018491954	273
ENSG00000241598	chr11	1642188	1643368	KRTAP5-4	gene	0.561244866	0.685189461	-0.123944594	0.123944594	0.01657164	383
ENSG00000198558	chr6	27840790	27842789	HIST1H4L	promoter	0.384314086	0.261793423	0.122520663	0.122520663	0.038509679	684
ENSG00000253128	chr14	107280753	107282752	IGHV4-80	promoter	0.421613585	0.544090353	-0.122476768	0.122476768	0.024707817	370
ENSG00000196867	chr19	57048817	57050816	ZFP28	promoter	0.180920404	0.303225677	-0.122305273	0.122305273	0.016197081	207
ENSG00000136275	chr7	47833389	47835388	C7orf69	promoter	0.440445774	0.318919304	0.12152647	0.12152647	0.005812677	108
ENSG00000170180	chr4	145030457	145061904	GYPA	gene	0.597327135	0.718728593	-0.121401459	0.121401459	0.023457271	509
ENSG00000199921	chr1	77217320	77219319	RNU6-161P	promoter	0.54671866	0.667822274	-0.121103614	0.121103614	0.005967944	703
ENSG00000233851	chr13	21577796	21579795	LATS2-AS1	promoter	0.678240846	0.55857641	0.119664437	0.119664437	0.017535475	756
ENSG00000196374	chr6	27782822	27783267	HIST1H2BM	gene	0.51287294	0.393260119	0.119612821	0.119612821	0.020334877	207
ENSG00000200089	chr14	101459573	101459647	SNORD114-31	gene	0.463432111	0.582935691	-0.11950358	0.11950358	0.002535141	237
ENSG00000271956	chr7	96635695	96637022	DLX6-AS2	gene	0.420360352	0.301817673	0.118542679	0.118542679	0.04220407	526
ENSG00000229473	chr13	41566915	41567467	RGS17P1	gene	0.497587125	0.379152821	0.118434303	0.118434303	0.016122591	156
ENSG00000229473	chr13	41566968	41568967	RGS17P1	promoter	0.497587125	0.379152821	0.118434303	0.118434303	0.016122591	209
ENSG00000204566	chr10	23528246	23530245	C10orf115	promoter	0.487921665	0.606108745	-0.11818708	0.11818708	0.001755841	802
ENSG00000236030	chr1	187060474	187062473	LINC01036	promoter	0.536635404	0.654260191	-0.117624786	0.117624786	0.013619739	666
ENSG00000250346	chr5	147300242	147302241	EEF1GP2	promoter	0.367752758	0.484759436	-0.117006678	0.117006678	0.018237802	257
ENSG00000250334	chr4	80413570	80497614	LINC00989	gene	0.341706315	0.457911655	-0.11620534	0.11620534	0.038836819	459
ENSG00000197706	chr12	55640982	55642086	OR6C74	gene	0.596026046	0.711694131	-0.115668084	0.115668084	0.004703461	581
ENSG00000173917	chr17	46622942	46624941	HOXB2	promoter	0.501000223	0.385353294	0.115646929	0.115646929	0.005761463	227
ENSG00000240443	chr12	48880229	48882228	RPS10P20	promoter	0.560122959	0.67558101	-0.115458051	0.115458051	0.036008803	886
ENSG00000110446	chr11	60719503	60721502	SLC15A3	promoter	0.39708904	0.512444468	-0.115355428	0.115355428	0.042635462	784

ENSG00000259954	chr16	27458990	27464714	IL21R-AS1	gene	0.379629801	0.494751542	-0.115121741	0.115121741	0.043882638	546
ENSG00000267961	chr19	15252350	15254349	OR10B1P	promoter	0.532943214	0.647183869	-0.114240654	0.114240654	0.036884615	779
ENSG00000207771	chr7	30327910	30329909	MIR550A1	promoter	0.603441739	0.489273036	0.114168703	0.114168703	0.00482488	597
ENSG00000180481	chr12	75783350	75785349	GLIPR1L2	promoter	0.17762824	0.291584616	-0.113956376	0.113956376	0.012373988	154
ENSG00000122861	chr10	75667435	75669434	PLAU	promoter	0.276981257	0.390699619	-0.113718362	0.113718362	0.042780431	792
ENSG00000240925	chr12	120685263	120685594	RPS20P31	gene	0.695800457	0.809302231	-0.113501773	0.113501773	0.000952198	980
ENSG00000240925	chr12	120683763	120685762	RPS20P31	promoter	0.695800457	0.809302231	-0.113501773	0.113501773	0.000952198	1881
ENSG00000125788	chr20	123010	126392	DEFB126	gene	0.474377959	0.587826309	-0.11344835	0.11344835	0.003422818	311
ENSG00000226263	chr20	13219822	13221821	ISM1-AS1	promoter	0.514841845	0.62788664	-0.113044795	0.113044795	0.026912319	736
ENSG00000266740	chr14	65801402	65803401	MIR4708	promoter	0.577366375	0.689865766	-0.112499392	0.112499392	0.005166572	1035
ENSG00000215024	chr10	48173347	48175106	DUSP8P2	gene	0.729473386	0.617716472	0.111756914	0.111756914	0.013949297	706
ENSG00000215024	chr10	48174607	48176606	DUSP8P2	promoter	0.729473386	0.617716472	0.111756914	0.111756914	0.013949297	1319
ENSG00000231209	chr10	48169665	48175810	GLUD1P6	gene	0.729473386	0.617716472	0.111756914	0.111756914	0.013949297	706
ENSG00000236675	chr1	155199266	155201265	MTX1P1	promoter	0.393866794	0.282841511	0.111025283	0.111025283	0.008892591	112
ENSG00000142327	chr2	241503721	241505720	RNPEPL1	promoter	0.675661947	0.78616388	-0.110501933	0.110501933	0.010223239	1868
ENSG00000200686	chr13	78928964	78930963	RNY3P3	promoter	0.551297945	0.66144209	-0.110144145	0.110144145	0.006893521	960
ENSG00000164256	chr5	23505764	23507763	PRDM9	promoter	0.538728778	0.647717621	-0.108988842	0.108988842	0.001654237	974
ENSG00000265402	chr18	49138981	49140980	RSL24D1P9	promoter	0.351576616	0.4603759	-0.108799283	0.108799283	0.030984313	492
ENSG00000094661	chr19	15197791	15199032	OR1I1	gene	0.688940306	0.796880915	-0.107940609	0.107940609	0.019012913	1092
ENSG00000252574	chr10	110944266	110946265	RNU5B-6P	promoter	0.499242837	0.606871718	-0.107628881	0.107628881	0.001536196	768
ENSG00000213871	chr3	25794916	25796915	TAF9BP1	promoter	0.623730285	0.731149549	-0.107419264	0.107419264	0.011723383	1553
ENSG00000242550	chr18	61562908	61564907	SERPINB10	promoter	0.582536377	0.689551084	-0.107014707	0.107014707	0.025926473	1250
ENSG00000230219	chr4	183958818	183961272	FAM92A1P2	gene	0.5521387	0.659041541	-0.106902841	0.106902841	0.041368103	586
ENSG00000263493	chr19	9934453	9936452	RN7SL94P	promoter	0.627719634	0.734235998	-0.106516364	0.106516364	0.029097385	1643
ENSG00000181867	chr5	121187650	121188519	FTMT	gene	0.642280266	0.748767308	-0.106487042	0.106487042	0.018918061	898
ENSG00000223015	chr11	87316175	87318174	RNU6-1135P	promoter	0.525452737	0.631144214	-0.105691476	0.105691476	0.006721969	816
ENSG00000129048	chr3	132314581	132316580	ACKR4	promoter	0.449442421	0.55487876	-0.10543634	0.10543634	0.007455565	590
ENSG00000146858	chr7	138720276	138722275	ZC3HAV1L	promoter	0.267711588	0.372976499	-0.105264911	0.105264911	0.046415575	903
ENSG00000233412	chr3	97886044	97888043	OR5H15	promoter	0.616084143	0.720995832	-0.104911689	0.104911689	0.013498763	1627
ENSG00000204148	chr9	118650448	118687486	LINC00474	gene	0.582667019	0.687062629	-0.10439561	0.10439561	0.014665401	740

ENSG00000213133	chr6	126579002	126581001	PPP1R14BP5	promoter	0.636166126	0.740538347	-0.104372221	0.104372221	0.008367786	1857
ENSG00000176742	chr11	5220965	5221930	OR51V1	gene	0.676248988	0.780586724	-0.104337736	0.104337736	0.006593816	1126
ENSG00000199812	chr3	146287764	146289763	RNU6-428P	promoter	0.664160341	0.768362115	-0.104201774	0.104201774	0.014853378	2159
ENSG00000263706	chr10	111762512	111764511	RN7SL450P	promoter	0.709761472	0.605672961	0.10408851	0.10408851	0.006581463	1573
ENSG00000207168	chr7	56128163	56128295	SNORA15	gene	0.527538669	0.631610331	-0.104071661	0.104071661	0.008922643	558
ENSG00000205181	chr20	5484760	5486759	LINC00654	promoter	0.425255408	0.529180657	-0.10392525	0.10392525	0.025118123	379
ENSG00000006377	chr7	96634860	96640351	DLX6	gene	0.477861042	0.37454295	0.103318092	0.103318092	0.045756194	576
ENSG00000236473	chr17	39604435	39610307	KRT43P	gene	0.60033028	0.702516649	-0.102186369	0.102186369	0.024182876	865
ENSG00000240470	chr15	90707965	90709964	RN7SL346P	promoter	0.314755362	0.416334119	-0.101578757	0.101578757	0.022619086	338
ENSG00000119125	chr9	74728011	74730010	GDA	promoter	0.562445202	0.664014891	-0.101569689	0.101569689	0.049381356	1338
ENSG00000269118	chr19	53811398	53813397	FAM90A28P	promoter	0.358355135	0.459650063	-0.101294928	0.101294928	0.017568397	241
ENSG00000160352	chr19	21263465	21265464	ZNF714	promoter	0.406164855	0.50717392	-0.101009064	0.101009064	0.043651694	824
ENSG00000203664	chr1	247654370	247655710	OR2W5	gene	0.574416687	0.674647729	-0.100231042	0.100231042	0.019611413	795
ENSG00000252695	chr13	24736555	24736643	MIR2276	gene	0.741681111	0.641579455	0.100101656	0.100101656	0.013806255	1104

Supplementary Table S3. GO enrichment analysis of top 45 hypermethylated pathways in PD-L1 high expressing glioblastomas. GO term enrichment analysis of top 45 hypermethylated pathways in PD-L1 high versus PD-L1 low expressing glioblastomas showed GO term enrichment of pathways being associated with post-transcriptional and RNA-associated gene regulation such as “DNA replication-dependent nucleosome assembly”, “chromatin silencing at rDNA”, and “DNA packaging” being hypermethylated in gene regions.

**PDL1 high versus low: Hypermethylated**						
**GOMFID**	**Pvalue**	**OddsRatio**	**ExpCount**	**Count**	**Size**	**Term**
GO:0006335	0	325.7763	0.3374	24	32	DNA replication-dependent nucleosome assembly
GO:0000183	0	186.0902	0.4007	24	38	chromatin silencing at rDNA
GO:0006323	0	27.8651	1.972	36	187	DNA packaging
GO:0051290	0	86.7579	0.5694	24	54	protein heterotetramerization
GO:0071824	0	18.7003	2.7418	36	260	protein-DNA complex subunit organization
GO:0016441	0	12.5303	4.6926	42	445	posttranscriptional gene silencing
GO:0045814	0	29.4472	1.3498	27	128	negative regulation of gene expression, epigenetic
GO:0031047	0	11.7519	4.9668	42	471	gene silencing by RNA
GO:0060968	0	23.7586	1.6029	27	152	regulation of gene silencing
GO:0045653	0	356.6975	0.1898	14	18	negative regulation of megakaryocyte differentiation
GO:0060964	0	29.8131	1.1705	24	111	regulation of gene silencing by miRNA
GO:0006333	0	27.0964	1.1392	22	117	chromatin assembly or disassembly
GO:0032200	0	17.7015	1.7927	24	170	telomere organization
GO:0034080	0	50.8827	0.4429	14	42	CENP-A containing nucleosome assembly
GO:0034724	0	37.4698	0.5484	14	52	DNA replication-independent nucleosome organization
GO:0016233	0	34.7218	0.58	14	55	telomere capping
GO:0030099	0	7.4802	4.2181	26	400	myeloid cell differentiation
GO:0038111	0	55.2075	0.2953	10	28	interleukin-7-mediated signaling pathway
GO:0006303	0	20.9009	0.8647	14	82	double-strand break repair via nonhomologous end joining
GO:0034622	0	4.5989	11.2201	41	1064	cellular protein-containing complex assembly
GO:0043044	0	20.0141	0.8963	14	85	ATP-dependent chromatin remodeling
GO:0045892	0	4.3963	12.0321	42	1141	negative regulation of transcription, DNA-templated

GO:1902679	0	4.2071	12.5172	42	1187	negative regulation of RNA biosynthetic process
GO:1903707	0	12.5061	1.5501	16	147	negative regulation of hemopoiesis
GO:0098760	0	36.7849	0.3902	10	37	response to interleukin-7
GO:0006334	0	17.381	0.8486	12	93	nucleosome assembly
GO:0045934	0	3.6492	14.6262	43	1387	negative regulation of nucleobase-containing compound metabolic process
GO:0051259	0	5.2173	5.8737	26	557	protein complex oligomerization
GO:0045652	0	22.1012	0.5733	10	59	regulation of megakaryocyte differentiation
GO:0009890	0	3.371	16.1342	44	1530	negative regulation of biosynthetic process
GO:0035195	0	7.1005	2.9613	18	323	gene silencing by miRNA
GO:0048534	0	3.7174	9.438	30	895	hematopoietic or lymphoid organ development
GO:0006352	0	6.8687	2.4887	15	236	DNA-templated transcription, initiation
GO:0045596	0	3.6431	7.4871	24	710	negative regulation of cell differentiation
GO:1903506	0	2.2154	34.9786	62	3317	regulation of nucleic acid-templated transcription
GO:0002227	0	28.3746	0.232	5	22	innate immune response in mucosa
GO:0006281	0	3.6115	5.5152	18	523	DNA repair
GO:2000112	0	2.021	39.0596	64	3704	regulation of cellular macromolecule biosynthetic process
GO:0051252	0	2.0259	37.5094	62	3557	regulation of RNA metabolic process
GO:0009954	0	18.5425	0.3269	5	31	proximal/distal pattern formation
GO:0050793	0	2.1101	26.7849	48	2540	regulation of developmental process
GO:0048731	0	1.895	48.9932	74	4646	system development
GO:0002251	0	15.5471	0.3796	5	36	organ or tissue specific immune response
GO:0019222	1.00E-04	1.8263	69.1029	95	6553	regulation of metabolic process
GO:0022607	1.00E-04	1.9869	30.8975	52	2930	cellular component assembly

Supplementary Table S4. GO enrichment analysis of top 45 hypomethylated pathways in PD-L1 high expressing glioblastomas. GO term enrichment analysis of top 45 hypomethylated pathways in PD-L1 high versus PD-L1 low expressing glioblastomas showed GO term enrichment of pathways being associated with post-transcriptional and RNA-associated gene regulation such as “posttranscriptional gene silencing by RNA”, “T-cell activation involved in immune response”, “leukocyte mediated cytotoxicity” and “cell killing”.

**PD-L1 high versus low: Hypomethylated**						
**GOMFID**	**Pvalue**	**OddsRatio**	**ExpCount**	**Count**	**Size**	**Term**
GO:0050911	0	19.1867	2.524	33	337	detection of chemical stimulus involved in sensory perception of smell
GO:0009593	0	15.702	3.1381	34	419	detection of chemical stimulus
GO:0007606	0	14.533	3.2355	33	432	sensory perception of chemical stimulus
GO:0050906	0	14.4219	3.2579	33	435	detection of stimulus involved in sensory perception
GO:0007186	0	5.9235	9.1147	39	1217	G protein-coupled receptor signaling pathway
GO:0050877	0	4.7036	9.7364	35	1300	nervous system process
GO:0009913	0	7.744	2.5989	17	347	epidermal cell differentiation
GO:0031424	0	16.1719	0.7383	10	102	keratinization
GO:0043588	0	6.104	3.0333	16	405	skin development
GO:0006069	1.00E-04	50.8924	0.0824	3	11	ethanol oxidation
GO:0051715	0.0015	44.8753	0.0599	2	8	cytolysis in other organism
GO:0070268	0.0015	6.4089	0.8388	5	112	cornification
GO:0042573	0.0016	14.5231	0.2322	3	31	retinoic acid metabolic process
GO:0061844	0.0018	8.3916	0.5168	4	69	antimicrobial humoral immune response mediated by antimicrobial peptide
GO:0045542	0.0019	38.4623	0.0674	2	9	positive regulation of cholesterol biosynthetic process
GO:0042572	0.0029	11.6136	0.2846	3	38	retinol metabolic process
GO:0016999	0.0041	5.071	1.0485	5	140	antibiotic metabolic process
GO:0010838	0.0041	24.4701	0.0974	2	13	positive regulation of keratinocyte proliferation
GO:0050830	0.0049	6.2612	0.6815	4	91	defense response to Gram-positive bacterium
GO:0009617	0.005	2.6499	4.4413	11	593	response to bacterium
GO:0060429	0.005	2.1122	9.287	18	1240	epithelium development
GO:0002475	0.0055	20.7029	0.1123	2	15	antigen processing and presentation via MHC class Ib
GO:0036166	0.0055	20.7029	0.1123	2	15	phenotypic switching

GO:0002824	0.0056	5.9846	0.7115	4	95	positive regulation of adaptive immune response based on somatic recombination of immune receptors built from immunoglobulin superfamily domains
GO:0035194	0.0062	2.8769	3.3253	9	444	posttranscriptional gene silencing by RNA
GO:0002286	0.0063	5.7925	0.734	4	98	T cell activation involved in immune response
GO:0097623	0.007	17.9404	0.1273	2	17	potassium ion export across plasma membrane
GO:1904753	0.007	17.9404	0.1273	2	17	negative regulation of vascular associated smooth muscle cell migration
GO:0001805	0.0075	Inf	0.0075	1	1	positive regulation of type III hypersensitivity
GO:0001812	0.0075	Inf	0.0075	1	1	positive regulation of type I hypersensitivity
GO:0002642	0.0075	Inf	0.0075	1	1	positive regulation of immunoglobulin biosynthetic process
GO:0032078	0.0075	Inf	0.0075	1	1	negative regulation of endodeoxyribonuclease activity
GO:0060118	0.0075	Inf	0.0075	1	1	vestibular receptor cell development
GO:0071329	0.0075	Inf	0.0075	1	1	cellular response to sucrose stimulus
GO:1901670	0.0075	Inf	0.0075	1	1	negative regulation of superoxide dismutase activity
GO:1904728	0.0075	Inf	0.0075	1	1	positive regulation of replicative senescence
GO:1904827	0.0075	Inf	0.0075	1	1	negative regulation of hydrogen sulfide biosynthetic process
GO:1904830	0.0075	Inf	0.0075	1	1	negative regulation of aortic smooth muscle cell differentiation
GO:1905955	0.0075	Inf	0.0075	1	1	negative regulation of endothelial tube morphogenesis
GO:0001909	0.0075	5.4983	0.7714	4	103	leukocyte mediated cytotoxicity
GO:0001906	0.0079	7.8732	0.4085	3	56	cell killing
GO:0018149	0.0085	7.661	0.4194	3	56	peptide cross-linking
GO:0071397	0.0088	15.8278	0.1423	2	19	cellular response to cholesterol
GO:0098581	0.0088	15.8278	0.1423	2	19	detection of external biotic stimulus
GO:0044364	0.0089	7.5187	0.4269	3	57	disruption of cells of other organism
